# Rectal Indomethacin Is Protective against Pancreatitis after Endoscopic Retrograde Cholangiopancreatography: Systematic Review and Meta-Analysis

**DOI:** 10.1155/2018/9784841

**Published:** 2018-05-09

**Authors:** Xingkang He, Wenfang Zheng, Yue Ding, Xia Tang, Jianmin Si, Lei-min Sun

**Affiliations:** ^1^Department of Gastroenterology, Sir Run Run Shaw Hospital, Zhejiang University Medical School, Hangzhou 310016, China; ^2^Institute of Gastroenterology, Zhejiang University (IGZJU), Hangzhou 310016, China; ^3^Department of Microbiology, Tumor and Cell Biology, Karolinska Institute, 171 77, Stockholm, Sweden; ^4^Sir Run Run Shaw Hospital, Zhejiang University Medical School, Hangzhou 310016, China

## Abstract

**Background and Aim:**

Rectal indomethacin was reported to be effective for postendoscopic retrograde cholangiopancreatography (ERCP) pancreatitis (PEP) prophylaxis. However, the preventive effect of indomethacin for average-risk patients remains unclear. Recently, some conflicting evidence was addressed by recent articles. We aimed to determine the protective role of indomethacin in PEP based on the latest available literature.

**Methods:**

A systematic literature search was conducted using PubMed, Embase, Web of Science, and the Cochrane Library to identify related articles published before October 2016. Studies that evaluated the administration of indomethacin in the prevention of PEP were included in the analysis. We adopted a random-effects model to calculate the overall relative risk (RR) and 95% confidence interval (CI).

**Results:**

Ten trials from an initial search were finally included in the meta-analysis. The administration of rectal indomethacin significantly reduced the incidence of PEP in consecutive ERCP population (RR, 0.63; 95% CI, 0.50–0.77). There was no significant heterogeneity across included studies (*I* ^2^ = 14.2%, *P* = 0.31). Further subgroup analyses also revealed that rectal indomethacin could protect the individuals at high and average risks and reduced severity of PEP. Pre-ERCP administration of indomethacin seemed to be better than the post-ERCP given. There was no evidence of significant publication bias.

**Conclusions:**

Rectal administration of indomethacin is an effective approach to prevent the incidence of PEP in both high- and average-risk populations undergoing ERCP. However, more high-quality RCTs are needed to further investigate the optimal timing for the administration of indomethacin.

## 1. Introduction

Post-ERCP pancreatitis (PEP) is a serious adverse event after ERCP, with a reported incidence of 9.7% in unselected patients [1]. Several risk factors were identified for PEP, such as “suspected sphincter of Oddi dysfunction” and “female gender” [2]. Given a huge economic and clinical burden, effective approaches for post-ERCP pancreatitis prophylaxis remain a major priority for research.

Nonsteroidal anti-inflammatory drugs (NSAIDs) are reported to be effective in PEP prophylaxis so far [3]. Several prospective RCTs and meta-analysis have well demonstrated that the rectal administration of indomethacin significantly decreased the rate of PEP [4–6]. Based on the above evidence, the European Society for Gastrointestinal Endoscopy (ESGE) guideline (2014) recommended the administration of 100 mg of rectal indomethacin for PEP prophylaxis in patients undergoing ERCP with no contraindication [3]. Subsequently, the Japanese Society of Hepato-Biliary-Pancreatic Surgery (2015) [7] also published similar guidelines. Indomethacin, therefore, as an effective pharmacologic prophylaxis, seemed to be appealing. In this context, some conflicting findings emerged recently. A recent prospective, double-blind, controlled trial conducted by Levenick et al. [8] in the USA showed that the reduction in PEP using indomethacin was not as significant as previously reported in multiple RCTs [4, 5, 9]. In fact, even more cases of pancreatitis occurred in the indomethacin group compared with the placebo group. Subsequently, a high-quality meta-analysis also concluded that there is no prophylaxis for the prevention of PEP among average-risk patients [10]. These findings raised the question of whether the administration of rectal indomethacin should be recommended in average-risk patients. However, a recent RCT with a large number of patients was performed in China and concluded that rectal indomethacin should be administrated across patients without contraindication prior to ERCP [11].

Therefore, the benefit of rectal indomethacin needs to be well demonstrated in the majority of patients (average-risk) undergoing ERCP in practice. Confronted with the above conflicting results, we performed a meta-analysis to assess the role of rectal indomethacin for PEP prophylaxis in average-risk individuals.

## 2. Methods

This systematic review and meta-analysis was conducted according to the Preferred Reporting Items of Systematic Reviews and Meta-Analyses (PRISMA) guidelines [12].

### 2.1. Literature Search

A comprehensive electric search was conducted across PubMed, Embase, and the Cochrane Library for relevant articles with no language limitations from database inception to October 2016. The following Medical Subject Heading (MeSH) terms and text words were adopted in the research: (‘indomethacin') AND (‘cholangiopancreatography endoscopic retrograde' OR ‘pancreatitis' OR ‘post-ERCP pancreatitis'). References of the included articles and reviews were also manually scrutinized.

### 2.2. Eligibility Criteria

The included criteria were based on the patients, intervention, comparator, outcomes, and study design (PICOS) criteria and were as follows[13]: (1) population: adults undergoing endoscopic retrograde cholangiopancreatography (ERCP); (2) intervention: assessment of rectal indomethacin prior to or post ERCP; (3) comparator: indomethacin exposure compared with placebo exposure or unexposed; (4) outcomes: risk of PEP, presented as relative risks (RR) with 95% confidence intervals (CI); and (5) study design: randomized controlled trial (RCT). Reviews, case reports, abstracts, and letters were excluded. Based on the above “PICOS” criteria, two reviewers (HXK and ZWF) independently scanned all titles and abstracts of articles after an initial search and removed apparently irrelevant studies. Afterward, we reviewed the full texts of the remaining articles in order to identify relevant studies for inclusion. When there were multiple publications from the same population, the most comprehensive article was included. Any discrepancies in the processes were discussed and resolved by agreement.

### 2.3. Definition of Patients and Outcomes

According to previous studies, the definition of PEP was referred by previous studies [5, 8, 9, 11, 14]. The severity was classified according to “the length of hospitalization and the degree of intervention required” [15]. The criteria to identify high- or average-risk individuals were based on the study by Elmunzer et al. [4]. Detailed information is summarized in Supplementary Data (available here) [4].

### 2.4. Data Extraction and Quality Assessment

Two authors (HXK and ZWF) independently extracted the related information from eligible articles regarding the author, year, study design, location, number of patients, intervention, and definition of PEP. The methodological quality of eligible studies was evaluated by two reviews using the Cochrane Collaboration's tool [16] independently. The tool included seven items for assessment, and each item was graded as low bias, high bias, or unclear. The discrepancy in data extraction and assessment was resolved by the third author (SLM).

### 2.5. Statistical Analysis

We adopted a random-effects model to calculate the overall estimate relative risk of PEP in relation to rectal indomethacin exposure with 95% confidence intervals (CI) considering the expected heterogeneity between studies. The heterogeneity among individual studies was assessed qualitatively by the Cochran *Q* statistic, with *P* < 0.1 indicating some heterogeneity [17]. The degree of heterogeneity was evaluated by *I* ^2^, and an *I* ^2^ > 30% suggests that there is moderate to high heterogeneity between included studies [17]. Furthermore, we carried out subgroup analyses stratified by selected population, the timing of administration, the severity of pancreatitis, and different regions. Sensitivity analyses were conducted by excluding each individual study in turn in order to ensure the robustness and consistency of overall results. The funnel plot asymmetry and Egger's test were performed to evaluate the potential publication bias. All statistical analyses were used by the Review Manager (RevMan) V.5.0 software and Stata version 13 (StataCorp, Texas, USA).

## 3. Results

### 3.1. Study Characteristics

**Figure 1:**
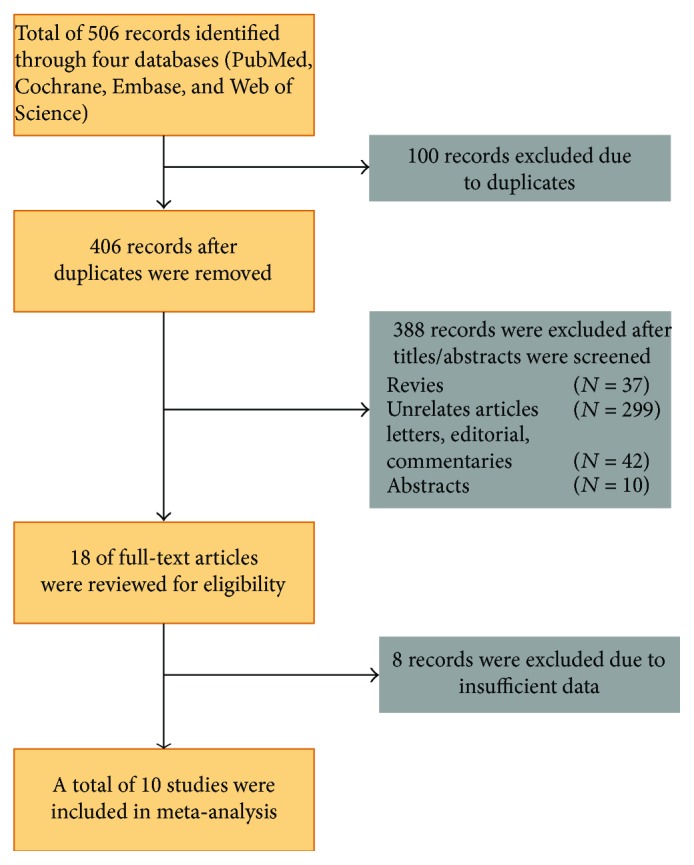
Flow diagram of included and excluded trials in this meta-analysis.

The initial search strategy yielded 506 studies, and ten studies [4, 5, 8, 9, 11, 14, 18–21] were finally included.  Figure 1 depicts the detailed selection and identification process. The characteristics of each individual study are summarized in Table 1. Overall, there was a total of 6094 patients undergoing ERCP, with 459 patients presenting with PEP. All trials adopted 100 mg rectal indomethacin suppository, whereas nine studies used placebo suppository as controls, and one study did not receive placebo. All trials adopted a similar definition of post-ERCP pancreatitis to include patients. The overall methodological quality of RCTs was generally moderate to high according to the Cochrane Collaboration's tool, and detailed information is shown in Supplementary Figures S1 and S2.

### 3.2. Overall Analyses of Rectal Indomethacin for PEP Prevention

**Figure 2:**
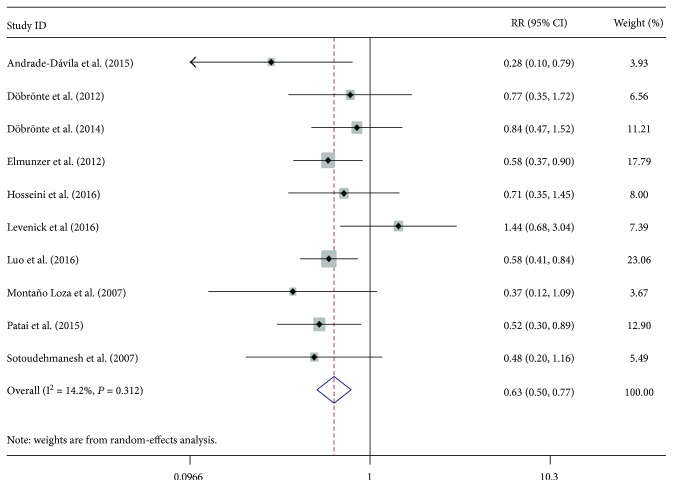
Forest plot for the overall relative risk of post-ERCP pancreatitis with rectal indomethacin.

A total ten of RCTs evaluated the prophylactic effect of rectal indomethacin on the prevention of PEP, with the incidence ranging from 4.83% to 13.66% [14, 20]. The relative risk (RR) of individual studies ranged from 0.28 to 1.44, and the cumulative meta-analysis by publication year showed that the rectal administration of indomethacin before or after ERCP was associated with a reduced risk of PEP in the overall population (RR = 0.63; 95% CI, 0.50–0.77) (Figure 2). Furthermore, we also performed a cumulative meta-analysis by publication year and number of included patients. The overall results gradually became stable and tended toward becoming significant with the increase in published year and larger samples (Supplementary Figures S3 and S4). The relatively low heterogeneity (*I* ^2^ = 14.2%, *P* = 0.31) was observed across included studies. Furthermore, sensitivity analyses by removing each study also supported the robustness of the overall outcomes in the meta-analysis. The funnel plot (Supplementary Figure S5) and Egger's test (*P* = 0.59) suggested no evidence of substantial publication bias in our analysis. No trial reported a higher incidence of adverse events associated with the administration of rectal indomethacin, suggesting the safeness of indomethacin.

### 3.3. Subgroup Analysis

#### 3.3.1. High-Risk versus Average-Risk Patients

**Figure 3:**
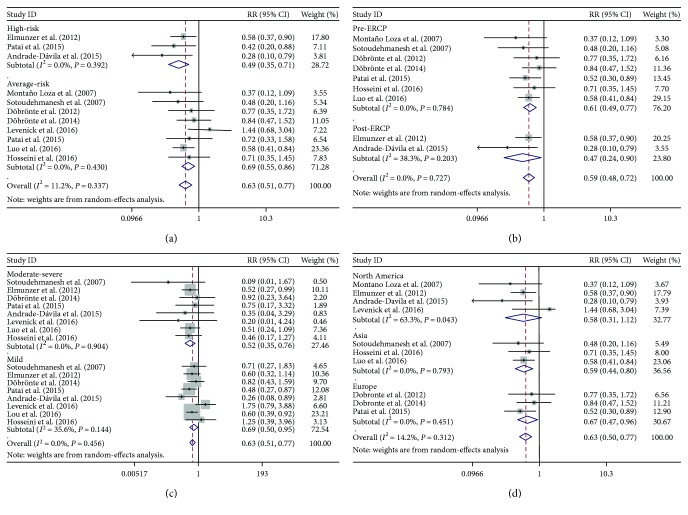
Forest plots of subgroup analysis stratified by (a) high-risk and average-risk patients, (b) pre-ERCP and post-ERCP administration, (c) mild and moderate-severe post-ERCP pancreatitis, and (d) patients from different regions.

Three studies selected high-risk population, and seven studies chose average-risk patients as the targeted population. The overall rates of PEP in high- and average-risk populations were 14.1% and 6.0%, respectively. The administration of indomethacin significantly reduced the risk of PEP among high-risk patients (RR, 0.49; 95% CI, 0.35–0.71), as well as across average-risk population (RR, 0.69; 95% CI, 0.55–0.86) (Figure 3(a)). No heterogeneity for average-risk and high-risk patients was noted. Furthermore, our sensitivity analyses also showed that the overall results were not changed by a single study.

#### 3.3.2. Pre-ERCP versus Post-ERCP Administration of Indomethacin

Most of the studies (7/10) administered indomethacin rectally prior to ERCP, two studies after ERCP, and one administered during ERCP. The pooled relative risks for pre- and post-ERCP administration were 0.61 (95% CI, 0.49–0.77) and 0.47 (95% CI, 0.24–0.90), respectively (Figure 3(b)). A low degree of heterogeneity (*I* ^2^ = 38.3%, *P* = 0.20) was noted among studies for post-ERCP while no heterogeneity existed in studies for pre-ERCP (*I* ^2^ = 0%, *P* = 0.78).

#### 3.3.3. Mild PEP versus Moderate-Severe PEP

Six Pooled studies showed that indomethacin administration significantly decreased the risk of mild and moderate-severe PEP (RR, 0.52, 95% CI, 0.35–0.76; RR = 0.69, 95% CI, 0.50–0.95, resp.) (Figure 3(c)). A relatively low heterogeneity (*I* ^2^ = 35.6%, *P* = 0.14) was observed across studies for mild PEP.

#### 3.3.4. Different Regions of Medical Centers

Among the included studies, three studies were performed in Asia (2/3 in Iran, 1/3 in China), three in Europe (3/3 in Hungary), and four in North America (2/4 in the United States, 2/4 in Mexico). The estimated pooled relative risks of PEP for Asia, Europe, and North America were 0.59 (95% CI, 0.44–0.80), 0.67 (95% CI, 0.47–0.96), and 0.58 (95% CI, 0.31–1.12), respectively. A high degree of heterogeneity (*I* ^2^ = 63.3%, *P* = 0.043) was noted among patients from North America.

## 4. Discussion

This exhaustive meta-analysis revealed a significant reduction of PEP risk (RR = 0.48, 95%, 0.26–0.87) in patients with rectal indomethacin. From 2006 to 2016, the cumulative meta-analysis by publication year showed that the overall result gradually became stable and tended toward becoming significant. In a subgroup analysis, the beneficial effect consistently favoured the administration of rectal indomethacin across most of the predefined variables. The prophylactic effect of rectal indomethacin was consistent across the average-risk and high-risk patients, and the administration of indomethacin before or after ERCP reduced the risk of mild and moderate-severe PEP. These results support the recommendation by ESGE and Japanese guidelines that individuals undergoing ERCP with no contraindications ought to be administrated indomethacin rectally to prevent post-ERCP pancreatitis [3, 7]. No increased risk of NSAID-associated adverse event was associated with the administration of indomethacin, indicating the safeness of rectal indomethacin.

Considering PEP as a serious adverse event, several pharmacologic agents were adopted for PEP prophylaxis, such as NSAIDs [22]. Several high-quality RCTs have demonstrated the effective prophylaxis of diclofenac and indomethacin for PEP, although the magnitude of benefits varied [3, 23]. In this context, it seemed that the rectal administration of NSAIDs is a sort of “panacea” for PEP prophylaxis [24]. However, discordant results from recent published RCTs and meta-analysis potentially challenge current evidence [8, 10]. The results of this trial showed that not only the effect of indomethacin for PEP was nonsignificant but also an opposite trend (higher incidence of pancreatitis in the indomethacin group than in the placebo group) was observed, although it is not significant. Levenick et al. [8] concluded that indomethacin may not prevent against PEP in ordinary population. However, we should interpret the conclusion with caution because the early termination of trial may lead to a type II statistical error [25]. Subsequently, a meta-analysis conducted by Inamdar et al. also showed that rectal indomethacin reduced the incidence of PEP in the high-risk patients, rather than in the average-risk [10] patients, which refuted the current guideline and previous meta-analysis. After that, two published RCTs also addressed this issue and supported the benefits of indomethacin in PEP prophylaxis.

Our findings are consistent with previous meta-analyses on this topic [6, 26, 27], although these are in contrast to a recent meta-analysis [10]. The advantage of the current study consists of the inclusion of enough high-quality RCTs, especially for some RCTs that have not been included in the previous studies. Relative low heterogeneity also reflects the similarity of included studies, which might further enhance the validity of results. Being different from the prior meta-analyses, our study is unique in performing cumulative meta-analyses and detailed subgroup analyses. However, this meta-analysis also has several limitations that merit further consideration. The final results and interpretations might be limited by the quantity and quality of included studies. Firstly, the significant heterogeneity across studies in North America could not be fully explained. Therefore, further trials should be conducted in the United States in order to examine the effect of indomethacin. Secondly, we were unable to identify clearly the optimal timing of administration due to lack of adequate RCT data. Finally, it was difficult to rule out the possibility of publication bias due to chance because of the limited number of included articles.

In summary, we demonstrated that rectal indomethacin significantly decreased PEP risk among high- or average-risk population undergoing ERCP and provided strong evidence for current guidelines in clinical practice. Considering its ease of administration, cost-effectiveness, and safety, indomethacin seemed to be an ideal and appealing pharmacological prophylaxis for PEP. However, optimal timing and its benefit in average-risk patients following ERCP needs to be confirmed in further larger prospective studies.

## Figures and Tables

**Table 1:** Characteristics of included studies in the meta-analysis.

Study	Year	Country	Type of trial	Patients (T/C)	Intervention	Definition of PEP
Sotoudehmanesh et al. [14]	2007	Iran	Double-blind randomized trial	245/245	100 mg rectal indomethacin versus inert suppository; before ERCP	Serum amylase more than 3 times the upper limit of normal associated with epigastric pain, back pain, and epigastric tenderness
Montaño Loza et al. [21]	2007	Mexico	Randomized controlled trial	75/75	100 mg rectal indomethacin versus rectal glycerine; before ERCP	Amylase level 3 times the upper limit of normal and epigastric pain or throughout the abdomen radiating to back associated with nausea or vomiting
Döbrönte et al. [19]	2012	Hungary	Prospective randomized clinical trial	130/98	100 mg rectal indomethacin versus inert placebo; before ERCP	Amylase level 3 times the upper limit of normal and epigastric pain or throughout the abdomen radiating to back associated with nausea or vomiting
Elmunzer et al. [4]	2012	American	Multicentre, randomized, placebo-controlled, double-blind clinical trial	295/307	2 ∗ 50 mg rectal indomethacin versus placebo suppository; after ERCP	Amylase level 3 times the upper limit of normal and epigastric pain or throughout the abdomen radiating to back associated with nausea or vomiting
Döbrönte et al. [18]	2014	Hungary	Multicentre prospective, randomized, controlled trial	347/318	100 mg, rectal indomethacin versus placebo suppository; before ERCP	Amylase level 3 times the upper limit of normal and epigastric pain or throughout the abdomen radiating to back associated with nausea or vomiting
Patai et al. [5]	2015	Hungary	Prospective, placebo-controlled, double-blind trial	270/269	100 mg rectal indomethacin versus placebo suppository; before ERCP	Abdominal pain, extended hospitalization 2–3 days, elevation of amylase 3 times the upper limit of normal in 24 hours
Andrade-Dávila et al. [9]	2015	Mexico	Prospective randomized controlled trial	82/84	100 mg rectal indomethacin versus glycerine; after ERCP	New or increased abdominal pain consistent with pancreatitis, elevated amylase or lipase greater than three times the normal upper limit until 24 hours after the procedure, and hospitalization (or prolongation of existing hospitalization) for at least 2 nights
Levenick et al. [8]	2016	America	Prospective, double-blind, placebo-controlledtrial	223/226	100 mg rectal indomethacin versus placebo suppository; during the ERCP	New upper abdominal pain, an elevated lipase greater than three times the upper limit of the normal 24 hours after the onset of pain, and hospitalization for at least two nights
Hosseini et al. [20]	2016	Iran	Randomized controlled trial	100/105	100 mg rectal indomethacin versus glycerine; before ERCP	New onset or worsened abdominal pain, increase in serum amylase at least 3 times above the upper limit of normal measured 24 h after the procedure, and need for more than one night of hospitalization
Luo et al. [11]	2016	China	Multicentre, single-blinded, randomized controlled trial	1297/1303	100 mg rectal indomethacin versus no treatment; before ERCP	New onset of upper abdominal pain associated with an elevated serum amylase of at least three times the upper limit of normal range at 24 h after the procedure and admission to a hospital for at least 2 nights

ERCP: endoscopic retrograde cholangiopancreatography; T/C: treatment/control.

## Abbreviations

ERCP: Endoscopic retrograde cholangiopancreatography
PEP: Postendoscopic retrograde cholangiopancreatography pancreatitis
RR: Relative risk
CI: Confidence interval
RCT: Randomized controlled trial
ESGE: European Society for Gastrointestinal Endoscopy
NSAIDs: Nonsteroidal anti-inflammatory drugs.

## Supplementary Materials

Supplementary Materials Supplementary Data: major and minor criteria for patients with high risk of post-ERCP pancreatitis. Supplementary Figure S1: quality assessments for included randomized control trials in the meta-analysis. Supplementary Figure S2: overall quality assessments for included randomized control trials in the meta-analysis. Supplementary Figure S3: cumulative meta-analysis of indomethacin for post-ERCP pancreatitis prevention by publication year. Supplementary Figure S4: cumulative meta-analysis of indomethacin for post-ERCP pancreatitis prevention by the number of patients. Supplementary Figure S5: funnel plot of all studies included in the present meta-analysis for the assessment of possible publication bias.

## References

[1] Elmunzer B. J. (2015).

[2] Dumonceau J. M., Andriulli A., Deviere J. (2010). European Society of Gastrointestinal Endoscopy (ESGE) Guideline: prophylaxis of post-ERCP pancreatitis.

[3] Dumonceau J. M., Andriulli A., Elmunzer B. J. (2014). Prophylaxis of post-ERCP pancreatitis: European Society of Gastrointestinal Endoscopy (ESGE) guideline – updated June 2014.

[4] Elmunzer B. J., Scheiman J. M., Lehman G. A. (2012). A randomized trial of rectal indomethacin to prevent post-ERCP pancreatitis.

[5] Patai A., Solymosi N., Patai A. V. (2015). Effect of rectal indomethacin for preventing post-ERCP pancreatitis depends on difficulties of cannulation: results from a randomized study with sequential biliary intubation.

[6] Yaghoobi M., Rolland S., Waschke K. A. (2013). Meta-analysis: rectal indomethacin for the prevention of post-ERCP pancreatitis.

[7] Yokoe M., Takada T., Mayumi T. (2015). Japanese guidelines for the management of acute pancreatitis: Japanese guidelines 2015.

[8] Levenick J. M., Gordon S. R., Fadden L. L. (2016). Rectal indomethacin does not prevent post-ERCP pancreatitis in consecutive patients.

[9] Andrade-Dávila V. F., Chávez-Tostado M., Dávalos-Cobián C. (2015). Rectal indomethacin versus placebo to reduce the incidence of pancreatitis after endoscopic retrograde cholangiopancreatography: results of a controlled clinical trial.

[10] Inamdar S., Han D., Passi M., Sejpal D. V., Trindade A. J. (2017). Rectal indomethacin is protective against post-ERCP pancreatitis in high-risk patients but not average-risk patients: a systematic review and meta-analysis.

[11] Luo H., Zhao L., Leung J. (2016). Routine pre-procedural rectal indometacin versus selective post-procedural rectal indometacin to prevent pancreatitis in patients undergoing endoscopic retrograde cholangiopancreatography: a multicentre, single-blinded, randomised controlled trial.

[12] Stewart L. A., Clarke M., Rovers M. (2015). Preferred Reporting Items for a Systematic Review and Meta-Analysis of individual participant data: the PRISMA-IPD Statement.

[13] Huang X., Lin J., Demner-Fushman D. (2006). Evaluation of PICO as a knowledge representation for clinical questions.

[14] Sotoudehmanesh R., Khatibian M., Kolahdoozan S., Ainechi S., Malboosbaf R., Nouraie M. (2007). Indomethacin may reduce the incidence and severity of acute pancreatitis after ERCP.

[15] Cotton P. B., Lehman G., Vennes J. (1991). Endoscopic sphincterotomy complications and their management: an attempt at consensus.

[16] Higgins J. P. T., Altman D. G., Gotzsche P. C. (2011). The Cochrane Collaboration’s tool for assessing risk of bias in randomised trials.

[17] Higgins J. P., Thompson S. G., Deeks J. J., Altman D. G. (2003). Measuring inconsistency in meta-analyses.

[18] Döbrönte Z., Szepes Z., Izbéki F. (2014). Is rectal indomethacin effective in preventing of post-endoscopic retrograde cholangiopancreatography pancreatitis?.

[19] Döbrönte Z., Toldy E., Márk L., Sarang K., Lakner L. (2012). Effects of rectal indomethacin in the prevention of post-ERCP pancreatitis.

[20] Hosseini M., Shalchiantabrizi P., Yektaroudy K., Dadgarmoghaddam M., Salari M. (2016). Prophylactic effect of rectal indomethacin administration, with and without intravenous hydration, on development of endoscopic retrograde cholangiopancreatography pancreatitis episodes: a randomized clinical trial.

[21] Montaño Loza A., Rodríguez Lomelí X., García Correa J. E. (2007). Effect of the administration of rectal indomethacin on amylase serum levels after endoscopic retrograde cholangiopancreatography, and its impact on the development of secondary pancreatitis episodes.

[22] Gu W. J., Liu J. C. (2013). Nonsteroidal anti-inflammatory drugs for prevention of post-ERCP pancreatitis: a complementary meta-analysis.

[23] Isaji S., Takada T., Mayumi T. (2015). Revised Japanese guidelines for the management of acute pancreatitis 2015: revised concepts and updated points.

[24] Freeman M. L., Kozarek R. A. (2016). Take 2 indomethacin (suppositories) and call me in the morning? The role of nonsteroidal anti-inflammatory drugs in protection against post-endoscopic retrograde cholangiopancreatography pancreatitis.

[25] Akshintala V. S., Singh V. K., Reddy D. N. (2016). Rectal indomethacin for post-ERCP pancreatitis prophylaxis in average risk patients: too early to terminate and too early to conclude.

[26] Ahmad D., Lopez K. T., Esmadi M. A. (2014). The effect of indomethacin in the prevention of post–endoscopic retrograde cholangiopancreatography pancreatitis: a meta-analysis.

[27] Shi N., Deng L., Altaf K., Huang W., Xue P., Xia Q. (2015). Rectal indomethacin for the prevention of post-ERCP pancreatitis: a meta-analysis of randomized controlled trials.

