# Supplementary material for: Rectal Indomethacin Is Protective against Pancreatitis after Endoscopic Retrograde Cholangiopancreatography: Systematic Review and Meta-Analysis
Source: Gastroenterol Res Pract. 2018 May 9;2018:9784841. doi: 10.1155/2018/9784841 (PMC5971281; doi:10.1155/2018/9784841)

**SUPPLEMENTARY DATA**

Major and minor criteria for patients with high risk of post-ERCP pancreatitis

| Major criteria | Minor criteria |
|---|---|
| clinical suspicion of sphincter of Oddi dysfunction, a history of PEP, pancreatic sphincterotomy, precut sphincterotomy, more than eight cannulation attempts, pneumatic dilatation of an intact biliary sphincter, or ampullectomy | age less than 50 years, female sex, a history of recurrent pancreatitis (2 episodes), three or more injections of contrast agent into the pancreatic duct with at least one injection to the tail of the pancreas, excessive injection of a contrast agent into the pancreatic duct resulting in opacification of pancreatic acini, the acquisition of a cytologic specimen from the pancreatic duct with the use of a brush |

## SUPPLEMENTARY FIGURE LEGEND

**Supplementary Figure 1.** Quality assessments for included randomized control trials in the meta-analysis

**Supplementary Figure 2.** Overall quality assessments for included randomized control trials in the meta-analysis

**Supplementary Figure 3.** Cumulative meta-analysis of indomethacin for post-ERCP pancreatitis prevention by publication year

**Supplementary Figure 4.** Cumulative meta-analysis of indomethacin for post-ERCP pancreatitis prevention by number of patients

**Supplementary Figure 5.** Funnel plot of all studies included in the present meta-analysis for assessment of possible publication bias.

**Supplementary Figure 1.**

| | Random sequence generation (selection bias) | Allocation concealment (selection bias) | Blinding of participants and personnel (performance bias) | Blinding of outcome assessment (detection bias) | Incomplete outcome data (attrition bias) | Selective reporting (reporting bias) | Other bias |
|---|---|---|---|---|---|---|---|
| Andrade-Davila 2015 | + | ? | + | + | + | + | + |
| Dobronte 2012 | + | ? | ? | ? | + | + | + |
| Dobronte 2014 | + | + | + | + | + | + | + |
| Elmunzer 2012 | + | + | + | + | + | – | + |
| Hosseini 2016 | + | + | + | + | + | + | + |
| Levenick 2016 | + | + | ? | – | + | + | + |
| Luo 2016 | + | + | ? | ? | + | + | + |
| Montano 2007 | + | ? | ? | – | + | + | + |
| Patai 2015 | + | + | + | + | + | – | – |
| Sotoudehmanesh 2007 | + | + | + | + | + | + | + |

* Green: Low risk, Yellow: Unclear, Red: High risk

**Supplementary Figure 2.**

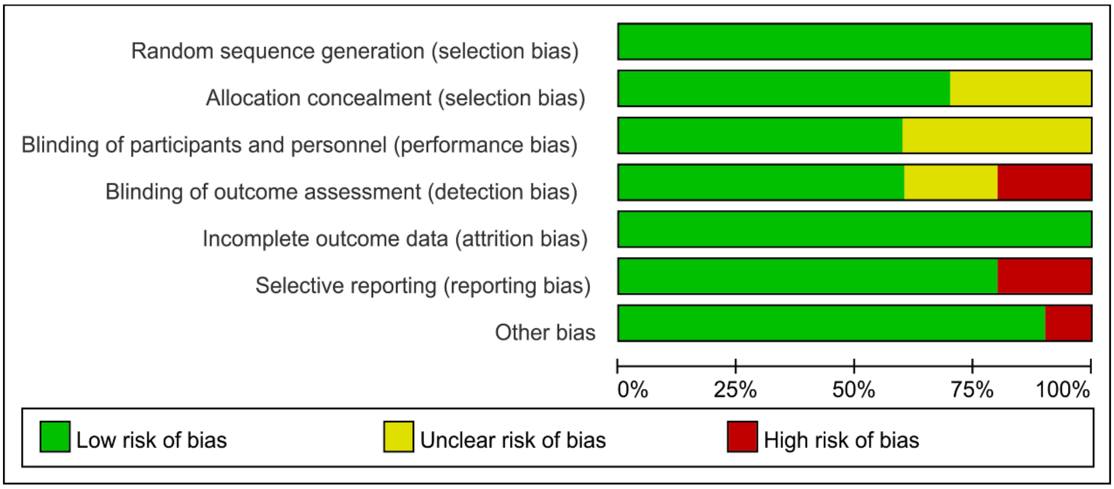

**Supplementary Figure 3.**

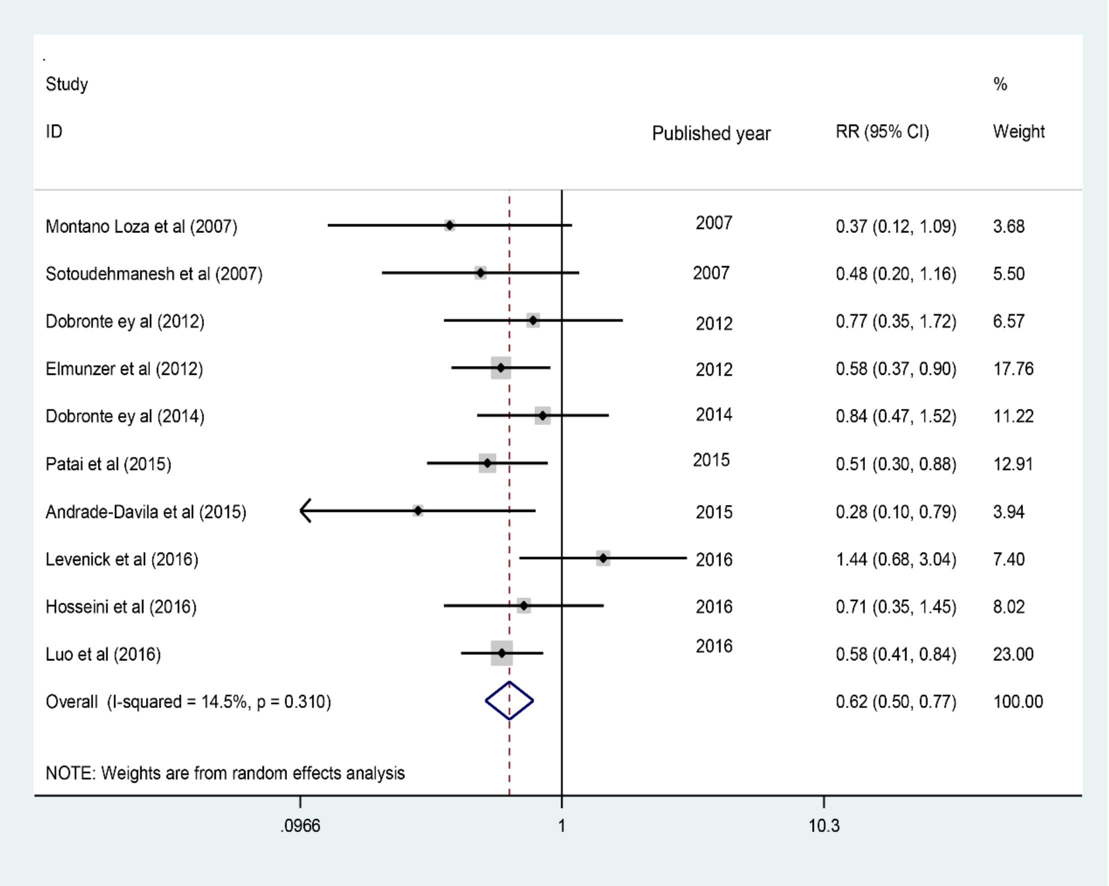

**Supplementary Figure 4.**

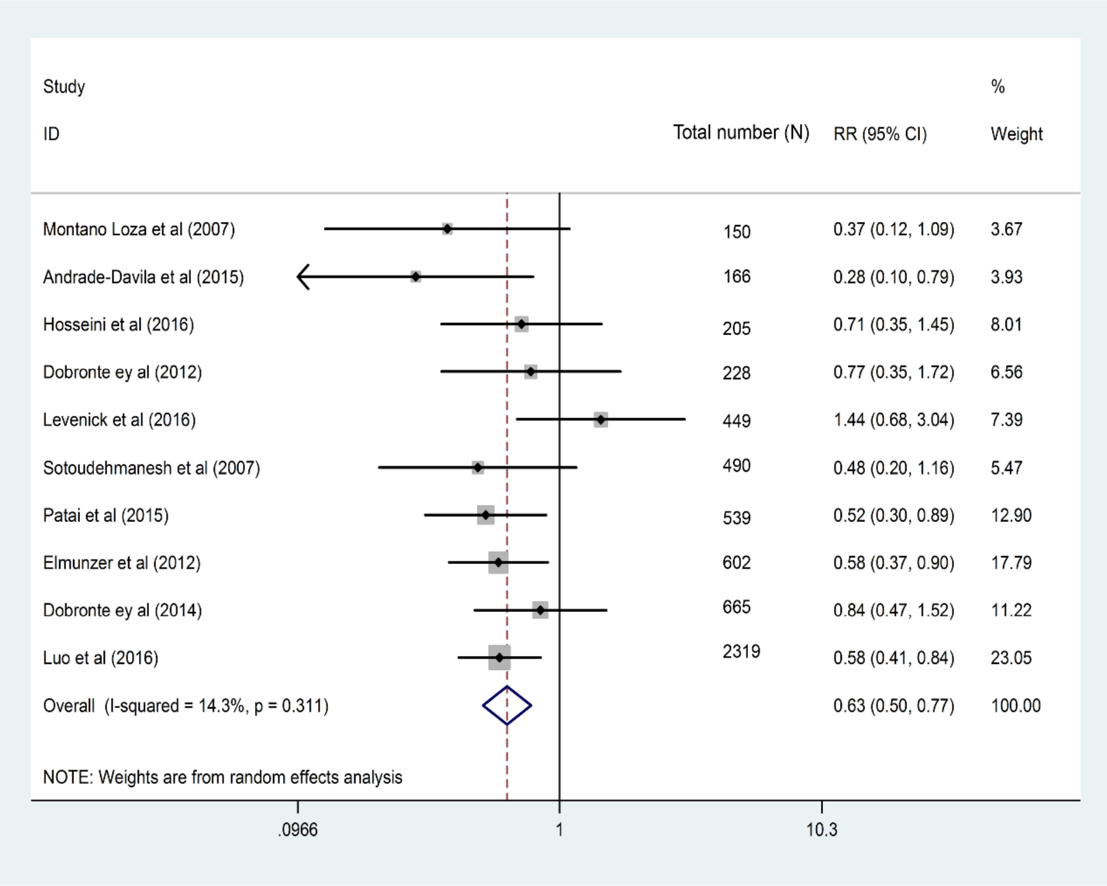

**Supplementary Figure 5.**

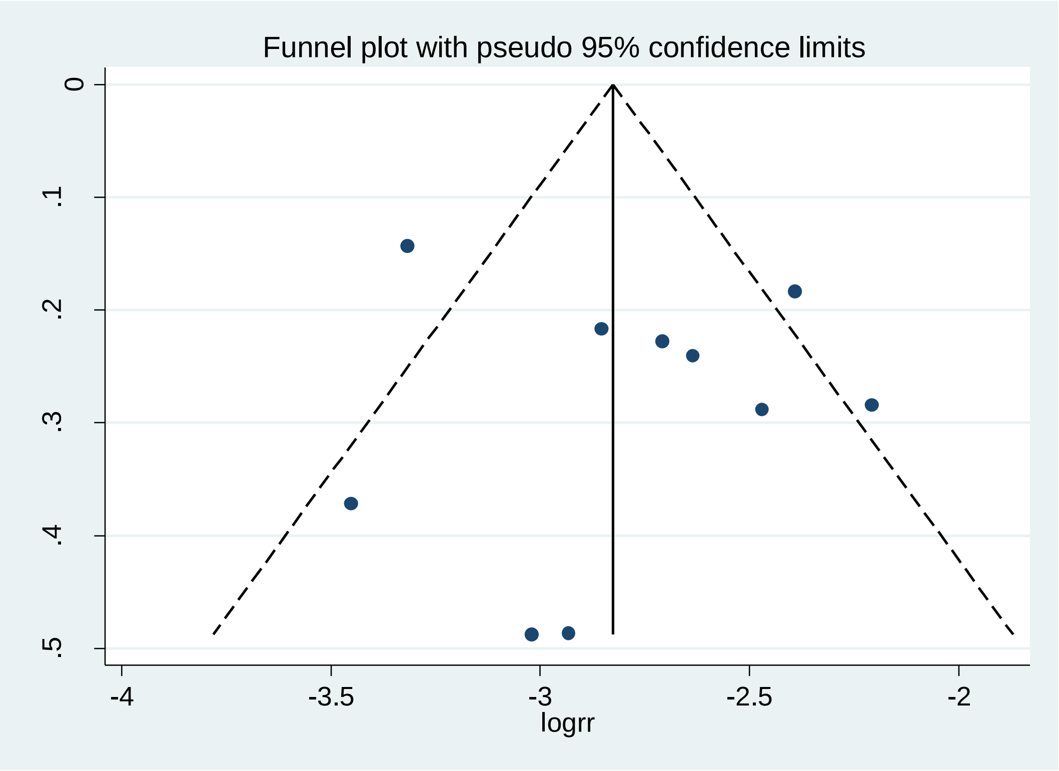

Supplement: Supplementary Materials — Supplementary Data: major and minor criteria for patients with high risk of post-ERCP pancreatitis. Supplementary Figure S1: quality assessments for included randomized control trials in the meta-analysis. Supplementary Figure S2: overall quality assessments for included randomized control trials in the meta-analysis. Supplementary Figure S3: cumulative meta-analysis of indomethacin for post-ERCP pancreatitis prevention by publication year. Supplementary Figure S4: cumulative meta-analysis of indomethacin for post-ERCP pancreatitis prevention by the number of patients. Supplementary Figure S5: funnel plot of all studies included in the present meta-analysis for the assessment of possible publication bias. [file 9784841.f1.pdf]
